# Prevalence, incidence and determinants of QuantiFERON^TM^ positivity in South African schoolchildren

**DOI:** 10.5588/ijtldopen.24.0084

**Published:** 2024-05-01

**Authors:** J. Stewart, N. Walker, K. Jennings, C. Delport, J Nuttall, A.K. Coussens, R Dyers, D.A. Jolliffe, J.C.Y. Tang, W.D. Fraser, R.J. Wilkinson, L.-G. Bekker, A.R. Martineau, K. Middelkoop

**Affiliations:** ^1^ Desmond Tutu HIV Centre, Department of Medicine, University of Cape Town, Cape Town, South Africa;; ^2^ Wolfson Institute of Population Health, Barts and The London School of Medicine and Dentistry, Queen Mary University of London, London, UK;; ^3^ Health Department, Cape Town Municipality, Cape Town,; ^4^ Paediatric Infectious Diseases Unit, Red Cross War Memorial Children's Hospital, Cape Town,; ^5^ Department of Paediatrics and Child Health, University of Cape Town, Rondebosch, Cape Town, South Africa;; ^6^ Infectious Diseases and Immune Defence Division, The Walter and Eliza Hall Institute of Medical Research, Parkville, VIC, Australia;; ^7^ Centre for Infectious Diseases Research in Africa, Institute of Infectious Disease and Molecular Medicine, University of Cape Town, Cape Town,; ^8^ Western Cape Government: Health and Wellness, Cape Town,; ^9^ Division of Health Systems and Public Health, Department of Global Health, Stellenbosch University, Stellenbosch, South Africa;; ^10^ Blizard Institute, Barts and The London School of Medicine and Dentistry, Queen Mary University of London, London,; ^11^ Norwich Medical School, University of East Anglia, Norwich Research Park, Norwich,; ^12^ Departments of Endocrinology and Clinical Biochemistry, Norfolk and Norwich University Hospitals Trust, Norwich,; ^13^ The Francis Crick Institute, London,; ^14^ Department of Infectious Diseases, Imperial College London, London, UK;; ^15^ Institute of Infectious Disease and Molecular Medicine, University of Cape Town, Cape Town, South Africa

**Keywords:** paediatric, latent TB, IGRA, QFT-Plus

## Abstract

**BACKGROUND:**

TB control requires the understanding and disruption of TB transmission. We describe prevalence, incidence and risk factors associated with childhood TB infection in Cape Town, South Africa.

**METHODS:**

We report cross-sectional baseline and prospective incidence data from a large trial among primary school children living in high TB burden communities. Prevalent infection was defined as QuantiFERON™-TB Gold Plus (QFT-Plus) positivity as assessed at baseline. Subsequent conversion to QFT-Plus positivity was measured 3 years later among those QFT-Plus-negative at baseline. Multivariable logistic regression models examined factors associated with TB infection.

**RESULTS:**

QuantiFERON-positivity at baseline (prevalence: 22.6%, 95% CI 20.9–24.4), was independently associated with increasing age (aOR 1.24 per additional year, 95% CI 1.15–1.34) and household exposure to TB during the participant’s lifetime (aOR 1.87, 95% CI 1.46–2.40). QFT-Plus conversion at year 3 (12.2%, 95% CI 10.5–14.0; annual infection rate: 3.95%) was associated with household exposure to an index TB case (aOR 2.74, 95% CI 1.05–7.18).

**CONCLUSION:**

Rates of QFT-diagnosed TB infection remain high in this population. The strong association with household TB exposure reinforces the importance of contact tracing, preventative treatment and early treatment of infectious disease to reduce community transmission.

## RÉSUMÉ

**CONTEXTE:**

La lutte contre la TB nécessite la compréhension et la perturbation de la transmission de la TB. Nous décrivons la prévalence, l'incidence et les facteurs de risque associés à l'infection tuberculeuse infantile au Cap, en Afrique du Sud.

**MÉTHODES:**

Nous rapportons des données transversales de référence et d'incidence prospective provenant d'un vaste essai mené auprès d'enfants d'écoles primaires vivant dans des communautés à forte charge de morbidité tuberculeuse. La prévalence de l'infection a été définie comme la positivité au QuantiFERON™-TB Gold Plus (QFT-Plus) telle qu'évaluée au départ. La conversion subséquente en QFT-Plus positif a été mesurée 3 ans plus tard chez les QFT-Plus négatifs au départ. Des modèles de régression logistique multivariée ont examiné les facteurs associés à l'infection tuberculeuse.

**RÉSULTATS:**

La positivité QuantiFERON-au départ (prévalence : 22,6%, IC à 95% 20,9–24,4), était indépendamment associée à l'augmentation de l'âge (aOR 1,24 par année supplémentaire, IC à 95% 1,15–1,34) et à l'exposition du ménage à la TB au cours de la vie du participant (aOR 1,87 ; IC à 95% 1,46–2,40). La conversion QFT-Plus à l'année 3 (12,2%, IC à 95% 10,5–14,0 ; taux d'infection annuel : 3,95%) était associée à l'exposition du ménage à un cas de tuberculose index (aOR 2,74 ; IC à 95% 1,05–7,18).

**CONCLUSION:**

Les taux d'infection tuberculeuse diagnostiquée par QFT restent élevés dans cette population. La forte association avec l'exposition à la TB dans les ménages renforce l'importance de la recherche des contacts, du traitement préventif et du traitement précoce des maladies infectieuses pour réduire la transmission communautaire.

Despite being treatable and curable, TB disease causes significant morbidity and mortality, impacting the lives of more than 10 million people every year, particularly in high-burden countries. Children under 15 years of age bear 11% of the global TB disease burden, with annual cases and deaths in this age group exceeding 1.1 million and 225,000, respectively.^1^

In high-burden countries *Mycobacterium tuberculosis* infection is often acquired in childhood.^2^ TB infection prevalence, based on measures of *M. tuberculosis* antigen immune memory, has been reported to range from 21% to 41% among primary school children in high-burden settings.^3–7^ A substantial reduction in the incidence and mortality of TB requires the interruption of *M. tuberculosis* transmission.^8^ Better understanding of the determinants of infection, would enable targeted medical, social and educational strategies to achieve TB control.

Factors positively associated with TB infection in high-burden settings include increasing age,^4,9–12^ male sex,^9–12^ known exposure to an infectious TB patient,^10,11,13^ low vitamin D status^9,14^ and exposure to secondhand smoke.^5,7,15^ There is inconsistency in risk factor findings between South African studies, such as the presence or absence of associations with socio-economic status^4,10^ and level of parental education.^4,10^ Given the high prevalence of TB in South Africa, we aimed to determine the prevalence and incidence of QuantiFERON-TB Gold Plus (QFT-Plus; Qiagen, Germantown, MD, USA) positivity as a measure of TB infection and identify associated risk factors among primary school children in Cape Town, South Africa.

## METHODS

This paper reports the analysis of cross-sectional baseline screening data and prospective incidence data from a large randomised placebo-controlled trial of vitamin D supplementation for the prevention of TB infection among primary school children living in Cape Town.^16^ The study was performed in parts of Khayelitsha, Klipfontein and Mitchells Plain districts of Cape Town. The community has substantial unemployment, with high-density, typically poorly constructed dwellings and relies predominantly on public health and education services. The incidence of active TB in this area is estimated at 799/100,000^17^ population per annum, as compared to the South African rate of 468/100,000.^18^

Recruitment and screening have been described previously.^19^ Potential participants were identified from 23 government-run primary schools within the districts described above. Eligibility criteria for the primary study included TB uninfected children (defined as a negative QFT-Plus interferon-gamma release assay [IGRA] result at screening), in grade 1 to 4 and aged 6–11 years at enrolment. Written consent was obtained from the parent or legal guardian, followed by assent granted by the children. Exclusion criteria included previous active TB, positive IGRA or Mantoux test and/or treatment thereof. Children with known or suspected HIV infection were also excluded from the study.

At screening visits, data were collected by interviewer-led questionnaire, including socio-economic characteristics and medical details including history of exposure to a household TB case. Following assent, the children were examined for features suggestive of TB disease and the presence of a bacille Calmette–Guérin (BCG) scar. Eligible children underwent phlebotomy for QFT-Plus blood assay and serum 25-hydroxy-vitamin D (25[OH]D_3_) concentrations. The QFT-Plus samples were collected according to manufacturer’s specifications and were delivered to the laboratory in temperature-controlled and monitored portable incubators (Model NQ09, Darwin Chambers Company, St Louis, MO, USA).^20^ Children testing QFT-Plus-positive or indeterminate were excluded from the prospective clinical trial following examination by a clinical team member for active TB. Eligible QFT-Plus-negative participants were enrolled and received vitamin D supplementation or placebo for up to three years, after which a repeat QFT-Plus assay was performed. Incident TB infection was defined as evidence of immune sensitisation indicated by QFT-Plus positive conversion at study completion.

### Laboratory methods

The latest generation IGRA was used to determine the outcome of interest. QFT-Plus has improved specificity and a lower indeterminate rate compared to the QuantiFERON-TB Gold In-Tube assay.^21^ QFT-Plus specimens were processed at Bio Analytical Research Corporation South Africa, a nationally accredited bioanalytical research laboratory. The assay was performed as per the manufacturers’ specifications in accordance with the US Food and Drug Administration package insert. The interferon-gamma threshold of 0.35 IU/ml was used to determine a positive result. Serum concentrations of 25[OH]D_3_ were determined using liquid chromatography–tandem mass spectrometry (LC-MS/MS)^19^ at a Bioanalytical Facility, University of East Anglia, Norwich, UK, as previously described.^22^

### Statistical analysis

Statistical analysis was performed using Stata software v17.0 (Stata Corp, College Station, TX, USA). Analysis was restricted to those with a valid QFT-Plus test result at the relevant time point. A conservative household crowding index was calculated based on the reported number of residents divided by the number of rooms in the house. Baseline (de-seasonalised) and Year 3 25(OH)D concentrations were used. For both prevalent and incident data, univariate analyses explored factors predictive of QFT positivity, employing Student’s *t*-tests and Wilcoxon rank-sum tests for continuous variables and χ^2^ and Fisher’s exact tests for categorical variables, as appropriate. Annual rate of infection- ARI, was estimated based on the QFT conversion rate at follow-up using the formula: ARI = 1−[(1−P)]1x, where *P* = proportion of QFT-positive individuals at follow-up (12.2%), *x* = the mean follow-up time for individuals who returned a QuantiFERON result (3.22 years). Multivariable logistic regression models were developed to examine factors associated with prevalent and incident TB infection at baseline and Year 3 respectively. Multivariable analysis included all independent variables with a *P*-value of less than 0.1 in univariable analysis and were adjusted for age and study arm allocation. Separate models were developed to evaluate characteristics of household contacts associated with prevalent QFT-Plus status. These models were adjusted for age and school district, as the two primary predictive factors identified for QFT-Plus positivity in the main analysis.

The study received approvals from the University of Cape Town Faculty of Health Sciences Human Research Ethics Committee, Cape Town, South Africa (Ref: 796/2015) and the London School of Hygiene & Tropical Medicine Observational/Interventions Research Ethics Committee, London, UK (Ref: 7450-2).

## RESULTS

### Determinants of prevalent TB infection

Parents of 2,852 children consented to their children’s study participation in the study (Figure). Overall, 40 (1.4%) children declined assent, or parents subsequently withdrew consent, and 211 (7.4%) children were ineligible. Reasons for ineligibility included challenges with phlebotomy procedures at baseline (*n* = 84, 2.9%), family planning to move out of the study area within the study period (*n* = 57, 2.0%) or history of previous latent or active TB (*n* = 55, 2.0%).

**Figure.**
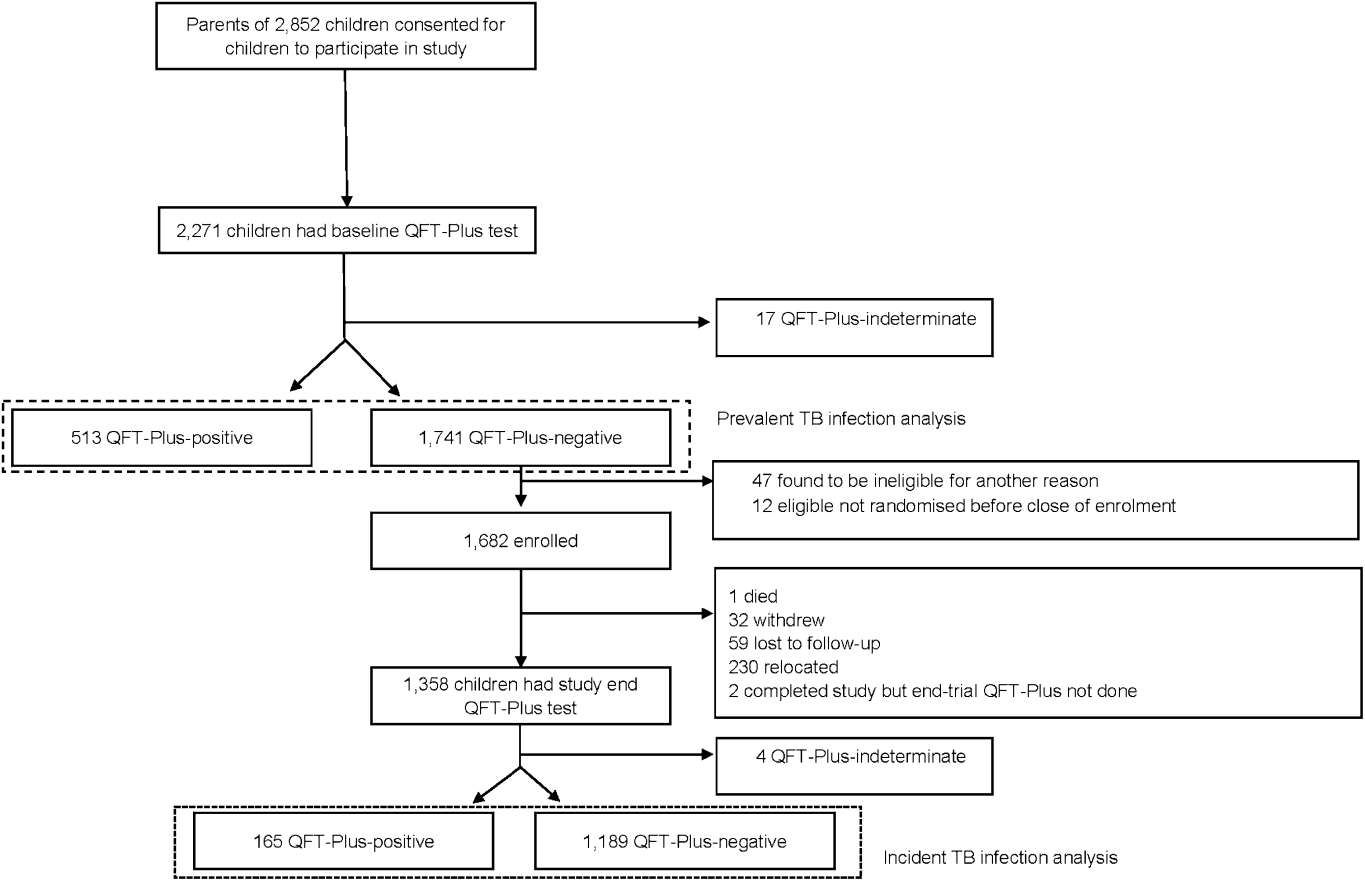
Participant flow. QFT-Plus = QuantiFERON-TB Gold Plus.

In total, 2,271 children had a screening QFT-Plus assay, with 17 (0.7%) producing an indeterminate result. The cohort characteristics of the 2,254 participants with valid QFT-Plus results are outlined in Table 1. This cohort had a median age of 9.1 years (interquartile range [IQR] 7.8–10.0); and 52.7% were female. Recruitment was spread throughout the study area with 800 (35.5%) participants from schools in Subdistrict 2, 387 (17.2%) from Subdistrict 5 and 355 (15.8%) from Subdistrict 1. The median monthly household income of the cohort was 1,200 South African rand - (IQR 700–2,500) with 52.4% living in informal housing. A total of 377 (16.6%) children had a history of reported exposure to one or more persons in their household living with pulmonary TB (PTB) during the child’s lifetime. Baseline serum 25(OH)D_3_ concentrations were available for 1,812 children; the mean concentration was 70.1 IU/ml (IQR 61.4–80.4) and 99 (5.5%) had a concentration of <50 nmol/L indicating vitamin D deficiency.^23^

**Table 1.** Characteristics of study participants included in analysis of determinants of baseline QFT-Plus status (*n* = 2,254).

Characteristic	*n* (%)
Demographic characteristics	
Age, years, median [IQR]	9.1 [7.8–10.0]
Sex	
Male	1,067 (47.3)
Female	1,187 (52.7)
Ethnicity	
IsiXhosa	2162 (97.7)
Coloured/mixed race	31 (1.4)
Other*	19 (0.9)
School subdistrict	
Subdistrict 1 (Mitchells Plain District)	355 (15.8)
Subdistrict 2 (Klipfontein District)	800 (35.5)
Subdistrict 3 (Mitchells Plain District)	273 (12.1)
Subdistrict 4 (Klipfontein District)	202 (8.9)
Subdistrict 5 (Khayelitsha District)	387 (17.2)
Subdistrict 6 (Mitchells Plain District)	237 (10.5)
Socio-economic and household characteristics	
Parental education^†‡^	
Primary school level	86 (3.8)
Secondary school education or higher	2,163 (96.0)
Monthly household income, ZAR, median [IQR]	1,200 [700–2,500]
Type of accommodation	
Brick	1,072 (47.6)
Informal	1,182 (52.4)
Number of people per room, median [IQR]	1.6 [1.3–2.5]
Anyone in the household smokes cigarettes indoors	322 (14.3)
Other potential risk factors/determinants	
BCG scar present	2,187 (97.2)
Number of people in the household diagnosed with pulmonary TB during the child’s lifetime	
0	1,877 (83.3)
1	342 (15.2)
2	32 (1.4)
3 or more	3 (0.1)
Season screened	
January–March	872 (38.7)
April–June	694 (30.8)
July–September	392 (17.4)
October–December	295 (13.1)
De-seasonalised serum 25(OH)D3 concentration, nmol/L, median [IQR]^‡^	70.1 [61.4–80.4]
<25	1 (0.1)
25–49.9	98 (5.4)
50–74.9	1,036 (57.2)
≥75	677 (37.4)

* Included Sothos, Vendas, Shonas, Swazis, Malawians and Zimbabweans.

† Highest level between both parents.

‡ Missing data: parental education level unknown (*n* = 5); serum 25(OH)D3 (*n* = 442).

QFT-Plus = QuantiFERON-TB Gold Plus; IQR = interquartile range; ZAR = South African rand; 25(OH)D3 = 25-hydroxyvitamin D₃.

The baseline prevalence of QFT-Plus positivity in this cohort was 22.6% (95% confidence interval [CI] 20.9–24.4). Factors associated with baseline QFT-Plus status are reported in Table 2. In univariate analysis, QFT-Plus-positive status was positively associated with increasing age (odds ratio [OR] 1.21 per year added, 95% CI 1.13–1.31), attending school in the Crossroads area (OR 1.91, 95% CI 1.32–2.77), living in informal housing (OR 1.25, 95% CI 1.03–1.52) and exposure to a household member living with PTB during the course of the participant’s lifetime (OR 1.93, 95% CI 1.52–2.46). Further, school attendance in Subdistrict 2 (OR 1.31, 95% CI 0.96–1.79) or the presence of anybody smoking cigarettes indoors trended towards a positive association with QFT-Plus positivity (OR 1.31, 95% CI 1.00–1.71). In multivariable regression (Table 2), there remained a statistically significant, positive association of prevalent TB infection with increasing age (adjusted OR [aOR] 1.24, 95% CI 1.15–1.34), school attendance in Subdistrict 3 (aOR 1.54, 95% CI 1.05–2.25) and household exposure to a person with PTB during the participant’s lifetime (aOR 1.87, 95% CI 1.46–2.40).

**Table 2.** Determinants of prevalent QFT-Plus positivity (*n* = 2,254).

Characteristic	QFT-Plus-positive *n/N* (%)	Univariate analysis		Multivariate analysis	
		OR (95% CI)	*P*-value	aOR (95% CI)	*P*-value
Demographics					
Age, years		1.21 (1.13 to 1.31)	<0.001*	1.24 (1.15 to 1.34)	<0.001*
6	43/259 (16.6)	Reference			
7	69/383 (18.0)	1.11 (0.73 to 1.68)	0.63		
8	91/411 (22.1)	1.44 (0.96 to 2.14)	0.08		
9	150/628 (23.9)	1.58 (1.08 to 2.30)	0.02*		
10	104/439 (23.7)	1.57 (1.06 to 2.23)	0.03*		
>11	56/132 (42.4)	3.67 (2.28 to 5.90)	<0.001		
Sex					
Male	237 (22.2)			Reference	
Female	276 (23.3)	1.06 (0.87 to 1.29)	0.56	1.08 (0.88 to 1.32)	0.45
Ethnicity					
IsiXhosa	491 (22.7)	Reference			
Coloured/other	13 (26.0)	1.20 (0.63 to 2.27)	0.58		
School subdistrict					
Subdistrict 1 (Mitchells Plain District)	66 (18.6)	Reference		Reference	
Subdistrict 2 (Klipfontein District)	184 (23.0)	1.31 (0.96 to 1.79)	0.09*	1.08 (0.78 to 1.49)	0.65
Subdistrict 3 (Mitchells Plain District)	83 (30.4)	1.91 (1.32 to 2.77)	0.001*	1.54 (1.05 to 2.25)	0.03*
Subdistrict 4 (Klipfontein District)	49 (24.3)	1.40 (0.92 to 2.13)	0.11	0.97 (0.63 to 1.51)	0.91
Subdistrict 5 (Khayelitsha District)	85 (22.0)	1.23 (0.86 to 1.77)	0.26	1.07 (0.74 to 1.55)	0.72
Subdistrict 6 (Mitchells Plain District)	46 (19.4)	1.05 (0.69 to 1.60)	0.80	0.71 (0.49 to 1.19)	0.24
Socio-economic and household characteristics					
Parental education^†‡^					
Primary school level	25 (29.1)	Reference			
Secondary school education or higher	488 (22.6)	0.71 (0.44 to 1.14)	0.16		
Monthly household income, ZAR, median [IQR]	1,200 [700–2,500]	1.00 (0.99 to 1.00)	0.45		
Type of accommodation					
Brick	222 (20.7)				
Informal	291 (24.6)	1.25 (1.03 to 1.52)*	0.03*	1.19 (0.97 to 1.47)	0.10
Number of people per room, median [IQR]	1.67 [1.25–2.5]	–0.01 (–0.2 to 0.01)	0.40		
Anyone in the household smokes cigarettes indoors	87 (27.0)	1.31 (1.00 to 1.71)*	0.05*	1.17 (0.88 to 1.54)	0.28
Other potential risk factors/determinants					
BCG scar present	498 (22.8)	1.15 (0.62 to 2.14)	0.64		
Exposed to household contact-diagnosed with pulmonary TB during the child’s lifetime	126 (33.4)	1.93 (1.52 to 2.46)	<0.001*	1.87 (1.46 to 2.40)	<0.001*
Season screened					
January–March	199 (22.8)	Reference			
April–June	152 (21.9)	0.95 (0.75 to 1.21)	0.67		
July–September	95 (24.2)	1.08 (0.82 to 1.43)	0.58		
October–December	67 (22.7)	0.99 (0.73 to 1.36)	0.97		
De-seasonalised serum 25(OH)D3 concentration, nmol/L, median [IQR]^‡^	70.2 [61.6–80.2]	1.00 (0.99 to 1.01)	0.60		
<25	0 (0)	Omitted^§^			
25–49.9	23 (23.5)	Reference			
50–74.9	223 (21.5)	0.89 (0.55 to 1.46)	0.66		
≥75	158 (23.3)	0.99 (0.60 to 1.64)	0.98		

* Statistically significant

† Highest level between both parents.

‡ Missing data: parental education level unknown (*n* = 5); serum 25(OH)D3 (*n* = 442).

§ Effect for default reference level inestimable as only one observation in this group.

QFT-Plus = QuantiFERON-TB Gold Plus; OR = odds ratio; CI = confidence interval; aOR = adjusted OR; IQR = interquartile range; ZAR = South African rand; 25(OH)D3 = 25-hydroxyvitamin D₃.

Among the 377 participants with a household PTB exposure in their lifetime, sleeping in the same room as the person with TB was the only statistically significant factor predictive of QFT-Plus positivity (Table 3). Neither time spent with the person with TB nor time from onset of cough to treatment initiation impacted participant QFT-Plus positivity.

**Table 3.** Determinants of infection risk in subset of participants reporting household exposure to pulmonary TB index case (*n* = 377).

Characteristics of household TB contact	QFT-Plus positivity *n/N* (%)	Univariate analysis*	
		OR (95% CI)	*P*-value
Number of people in the household diagnosed with pulmonary TB during the child’s lifetime			
1	115/342 (33.6)	Reference	
2	10/32 (31.3)	0.93 (0.42–2.07)	0.87
3	1/3 (33.3)	0.96 (0.08–10.84)	0.97
Time since child’s last contact with index case, years, median [IQR]	1.26 [0–3.9]	0.97 (0.89–1.05)	0.39
Child’s proximity to index case			
Sleeps in a different room	51/183 (27.9)	Reference	
Sleeps in the same room	75/194 (38.7)	1.79 (1.14–2.82)	0.01^†^
Time child spent with index case			
Not every day	30/104 (28.9)	Reference	
Every day, ≥50% of time	51/131 (38.9)	1.58 (0.90–2.77)	0.11
Every day, <50% of time	45/142 (31.7)	1.15 (0.66–2.03)	0.62
How long did index case cough before TB treatment was started, weeks			
≤1	12/35 (34.3)	Reference	
2	64/205 (31.2)	1.0 (0.46–2.17)	0.99
3	32/90 (35.6)	1.16 (0.50–2.69)	0.72
4	14/32 (43.8)	1.64 (0.60–4.47)	0.33
≥5	4/15 (26.7)	0.78 (0.20–3.04)	0.72

* Adjusted for age and school subdistrict as factors, other than household contact, significantly associated with positive QFT-Plus.

† Statistically significant.

QFT-Plus = QuantiFERON-TB Gold Plus; OR = odds ratio; CI = confidence interval; IQR = interquartile range.

### Determinants of TB infection at study end

In the prospective analysis, among the 1,354 children who were QFT-Plus-negative at baseline and who had a valid QFT-Plus results at the end of the study, 165 (12.2%, 95% CI 10.5–14.0) had a positive end-study QFT-Plus result. This equated to an annual infection rate of 3.95% (95% CI 3.36–4.54). In both univariate and multivariate analysis of incident QFT-Plus positivity at the end of the study, exposure to a household TB contact over the 3 years of study participation was the only independently associated predictor (aOR 2.74, 95% CI 1.05–7.18; Table 4). Female sex trended towards a significant association with incident QFT-Plus positivity in both univariate (*P* = 0.08) and multivariate analysis (*P* = 0.05). Factors associated with prevalent TB infection, such as age and area of school attendance, were not found to be associated with incident infection in univariate analysis (*P* = 0.13).

**Table 4.** Determinants of incident QFT-Plus positivity (*n* = 1,354).

Characteristic	QFT-Plus positivity at 3-year follow-up *n/N* (%)	Univariate analysis		Multivariate analysis	
		OR (95% CI)	*P*-value	aOR (95% CI)	*P*-value
Demographics					
Age at study end, years		1.04 (0.91 to 1.19)	0.56	1.05 (0.91 to 1.20)	0.51
9	10/91 (11.0)				
10	31/232 (13.4)				
11	29/285 (10.2)				
12	53/429 (12.4)				
13	34/255 (13.3)				
14	8/62 (12.9)				
Sex					
Male	66/633 (10.4)	Reference		1	
Female	99/719 (13.8)	1.36 (0.97 to 1.90)	0.07	1.40 (1.00 to 1.96)	0.05
Ethnicity					
IsiXhosa	161/1300 (12.4)	Reference			
Coloured/Other	2/29 (6.9)	0.56 (0.13 to 2.44)	0.44		
School subdistrict					
Subdistrict 1 (Mitchells Plain District)	20/196 (10.2)	Reference			
Subdistrict 2 (Klipfontein District)	56/501 (11.2)	1.12 (0.62 to 2.02)	0.70		
Subdistrict 3 (Mitchells Plain District)	20/153 (13.1)	1.33 (0.65 to 2.69)	0.43		
Subdistrict 4 (Klipfontein District)	8/129 (6.2)	0.59 (0.24 to 1.44)	0.25		
Subdistrict 5 (Khayelitsha District)	37/230 (16.1)	1.66 (0.87 to 3.18)	0.13		
Subdistrict 6 (Mitchells Plain District)	24/145 (16.6)	1.74 (0.87 to 3.46)	0.11		
Socio-economic and household characteristics					
Parental education (baseline)*					
Primary school level	7/46 (15.2)	1			
Secondary school education or higher	156/1305 (11.9)	0.74 (0.32 to 1.71)	0.48		
Monthly household income, ZAR, median [IQR]	1,200 (700 to 2,500)	0.99 (0.92 to 1.07)	0.89		
Type of accommodation					
Brick	93/731 (12.7)	1			
Informal	72/623 (11.6)	0.87 (0.61 to 1.22)	0.40		
Number of people per room, median [IQR]	1.67 (1.3 to 2.5)	0.99 (0.87 to 1.12)	0.88		
Anyone in the household smokes cigarettes indoors				
No	138/1169 (11.8)	1			
Yes	27/185 (14.6)	1.27 (0.81 to 1.96)	0.34		
Other potential risk factors/determinants					
BMI *Z*-score, median [IQR]^†^	0.21 (–0.6 to 1.0)	1.01 (0.96 to 1.06)	0.77		
Exposed to household contact- diagnosed with pulmonary TB during the past 3 years					
No	159/1,328 (11.9)	1		1	
Yes	6/25 (24.0)	2.64 (1.02 to 6.90)	0.05	2.74 (1.05 to 7.18)	0.04
Season end of study sample collected					
January-March	12/130 (9.23)	1.00			
April-June	56/477 (11.74)	1.28 (0.63 to 2.60)			
July-September	56/393 (14.25)	1.49 (0.72 to 3.06)			
October-December	41/354 (11.58)	1.25 (0.61 to 2.59)	0.70		
Serum 25(OH)D3 concentration, median [IQR]					
Baseline	70.04 (61.32 to 80.37)	1.01 (1.00 to 1.02)	0.25		
Year 3	79.15 (63.20 to 102.80)	1.00 (0.99 to 1.00)	0.36		
Time from baseline to QFT assessment (time on study), years	3.14 (2.96 to 3.28)	0.53 (0.23 to 1.23)	0.14		
Study arm allocation					
Control	89/687 (12.95)	1		1	
Intervention	76/667 (11.39)	0.86 (0.62 to 1.19)	0.35	0.86 (0.61 to 1.19)	0.36

* Highest level between both parents.

† ORs reflects data on continuous scale.

QFT-Plus = QuantiFERON-TB Gold Plus; OR = odds ratio; CI = confidence interval; aOR = adjusted OR; ZAR = South African rand; IQR = interquartile range; BMI = body mass index; 25(OH)D3 = 25-hydroxyvitamin D₃.

## DISCUSSION

This study reports a high prevalence and incidence of TB infection, defined as immune sensitisation determined by QFT-Plus positivity, among primary school children in a high TB burden setting. The prevalence of 22.6% (95% CI 20.9–24.4) is consistent with previously published data from similar demographic areas in Cape Town.^3,10^ However, this is the first report of such data from the populous Klipfontein and Mitchells Plain Districts, representing the most recent sub-Saharan TB infection prevalence data using the improved diagnostic assay of QFT-Plus. Furthermore, 12% of children converted to QFT-Plus positivity, equating to a minimum ARI of 3.95%, measured across the 3-year follow-up period.

Key determinants of prevalent QFT-diagnosed TB infection in this study included increasing age, exposure to at least one person with PTB living in the same household over the lifetime of the child and the subdistrict in which the child attended school. Increasing age and household TB exposure are established risk factors for acquisition of TB infection.^4,9–11,13^ Among participants who had lived with at least one person with PTB, only sleeping in the same room was statistically associated with prevalent TB infection, suggestive of a likely transmission link. Other anticipated factors, notably the number of persons who had TB in the house and time spent with those with TB were not associated with baseline QFT-Plus positivity. This may be due to the small number of participants included in this sub analysis, reducing power to detect associations.

Given limited scholar transport options in this district, the subdistrict of school attendance is a reasonable surrogate for the participants’ residential area. While particular subdistricts were more strongly associated with prevalent TB infection, there was no association between school subdistrict and incident infection. The association with prevalent TB infection may indicate historical differences between subdistricts such as variable timing of demographic and socio-economic expansion. However, the incident data suggests that the risk across areas is now homogenous.

In contrast to data recently published from a Mongolian cohort,^9^ no association was identified between QFT-Plus positivity and vitamin D status or indoor cigarette smoking at home. This may be explained by the very discrepant prevalence of these factors between the populations: the South African cohort had an overall prevalence of vitamin D deficiency (25[OH]D <50 nmol/L) of 7.6%,^19^ compared to the Mongolian cohort prevalence of 95.6%.^9^ This discrepancy is likely due to factors such as differing sun exposure and diet.^19^ Similarly, while smoking is a well-recognised risk factor for TB infection,^15,24,25^ only 14.3% of parents reported that a household member smoked indoors, compared to 36.3% in the Mongolian study. The nature of exposure to cigarette smoke may also be different across the two settings: given the milder winters in Cape Town, smokers may be able to smoke outdoors more often when compared to smokers based in Mongolia with its more prolonged inclement winters.

The only measured factor associated with incident TB infection was exposure to a household TB case. Female participants trended towards a higher odds of incident infection compared to male participants. This is in keeping with other reports for this young adolescent age group.^26^ This finding should be explored further in larger studies, together with possible explanations, such as different social mixing patterns between the sexes, or whether females spent more time in the household with a possible TB case.^27,28^ The lack of association with the pharmacological effect of vitamin D supplementation in this clinical trial is an important finding, which aligns with previous reports,^16^ and is consistent with other recent studies in young children.^29^

Key strengths of this study include the combination of cross-sectional and prospective data, enabling a rare opportunity to compare prevalent and incident TB infection risk factors in the same population, and the large sample of young school children. Schooling is compulsory in the age group studied and the implementation of this study across 23 government schools provided wide community representation, thus ensuring reasonable population generalisability. This study contributes to the few data evaluating IGRA testing as a marker for TB infection in a 6–14 year old paediatric population. The assay used was the latest generation of QFT tests, with improved diagnostic parameters and was performed by an accredited laboratory, with <1% of samples reported as ‘indeterminate’. Further, although the subset of participants reporting household exposure to a household person with PTB was relatively small, we obtained detailed information regarding household TB exposure, such as sleeping arrangements and the time spent together.

It is noted that this study sample is not a randomly selected cohort, but a screening cohort for a randomised placebo-controlled trial. This study cohort is largely limited to black South African children and is therefore not representative of all population groups. The association between prevalent TB infection and area of school attendance assumes that children attend school in their residential area, which is reasonable based on low economic resources and limited scholar transport options in the area. Furthermore, body mass index, a common factor associated with TB infection, could not be included in the prevalent infection analysis as these data were collected only on QFT-negative children eligible for the primary study.

In conclusion, TB infection remains a substantial burden in this setting. Our study adds original data from South Africa reporting a high prevalence and incidence of QFT-Plus diagnosed TB infection among the predominantly black South African primary school children enrolled in this study. The strong association with TB exposure in a household reinforces the importance of current WHO recommendations for contact tracing and early initiation of preventive or treatment regimens for all household contacts in high TB burden settings to reduce household and community transmission of TB by averting or disrupting individual infectiousness.
